# Author Correction: Attenuated fusogenicity and pathogenicity of SARS-CoV-2 Omicron variant

**DOI:** 10.1038/s41586-026-10665-7

**Published:** 2026-05-29

**Authors:** Rigel Suzuki, Daichi Yamasoba, Izumi Kimura, Lei Wang, Mai Kishimoto, Jumpei Ito, Yuhei Morioka, Naganori Nao, Hesham Nasser, Keiya Uriu, Yusuke Kosugi, Masumi Tsuda, Yasuko Orba, Michihito Sasaki, Ryo Shimizu, Ryoko Kawabata, Kumiko Yoshimatsu, Hiroyuki Asakura, Mami Nagashima, Kenji Sadamasu, Kazuhisa Yoshimura, The Genotype to Phenotype Japan (G2P-Japan) Consortium, Mai Suganami, Akiko Oide, Mika Chiba, Hayato Ito, Tomokazu Tamura, Kana Tsushima, Haruko Kubo, Zannatul Ferdous, Hiromi Mouri, Miki Iida, Keiko Kasahara, Koshiro Tabata, Mariko Ishizuka, Asako Shigeno, Kenzo Tokunaga, Seiya Ozono, Isao Yoshida, So Nakagawa, Jiaqi Wu, Miyoko Takahashi, Atsushi Kaneda, Motoaki Seki, Ryoji Fujiki, Bahityar Rahmutulla Nawai, Yutaka Suzuki, Yukie Kashima, Kazumi Abe, Kiyomi Imamura, Kotaro Shirakawa, Akifumi Takaori-Kondo, Yasuhiro Kazuma, Ryosuke Nomura, Yoshihito Horisawa, Kayoko Nagata, Yugo Kawai, Yohei Yanagida, Yusuke Tashiro, Otowa Takahashi, Kazuko Kitazato, Haruyo Hasebe, Chihiro Motozono, Mako Toyoda, Toong Seng Tan, Isaac Ngare, Takamasa Ueno, Akatsuki Saito, Erika P. Butlertanaka, Yuri L. Tanaka, Nanami Morizako, Hirofumi Sawa, Terumasa Ikeda, Takashi Irie, Keita Matsuno, Shinya Tanaka, Takasuke Fukuhara, Kei Sato

**Affiliations:** 1 Department of Microbiology and Immunology, Graduate School of Medicine, Hokkaido University, Sapporo, Japan; 2 Division of Systems Virology, Department of Infectious Disease Control, International Research Center for Infectious Diseases, The Institute of Medical Science, The University of Tokyo, Tokyo, Japan; 3 Faculty of Medicine, Kobe University, Kobe, Japan; 4 Department of Cancer Pathology, Faculty of Medicine, Hokkaido University, Sapporo, Japan; 5 Institute for Chemical Reaction Design and Discovery (WPI-ICReDD), Hokkaido University, Sapporo, Japan; 6 Division of Molecular Pathobiology, International Institute for Zoonosis Control, Hokkaido University, Sapporo, Japan; 7 Division of International Research Promotion, International Institute for Zoonosis Control, Hokkaido University, Sapporo, Japan; 8 One Health Research Center, Hokkaido University, Sapporo, Japan; 9 Division of Molecular Virology and Genetics, Joint Research Center for Human Retrovirus infection, Kumamoto University, Kumamoto, Japan; 10 Department of Clinical Pathology, Faculty of Medicine, Suez Canal University, Ismailia, Egypt; 11 Graduate School of Medicine, The University of Tokyo, Tokyo, Japan; 12 Laboratory of Systems Virology, Institute for Frontier Life and Medical Sciences, Kyoto University, Kyoto, Japan; 13 Graduate School of Pharmaceutical Sciences, Kyoto University, Kyoto, Japan; 14 International Collaboration Unit, International Institute for Zoonosis Control, Hokkaido University, Sapporo, Japan; 15 Institute of Biomedical and Health Sciences, Hiroshima University, Hiroshima, Japan; 16 Institute for Genetic Medicine, Hokkaido University, Sapporo, Japan; 17 Tokyo Metropolitan Institute of Public Health, Tokyo, Japan; 18 Division of Risk Analysis and Management, International Institute for Zoonosis Control, Hokkaido University, Sapporo, Japan; 19 CREST, Japan Science and Technology Agency, Saitama, Japan; 20 National Institute of Infectious Diseases, Tokyo, Japan; 21 Tokai University, Isehara, Japan; 22 Chiba University, Chiba, Japan; 23 The University of Tokyo, Kashiwa, Japan; 24 Kyoto University, Kyoto, Japan; 25 Kumamoto University, Kumamoto, Japan; 26 University of Miyazaki, Miyazaki, Japan

**Keywords:** SARS-CoV-2, Viral pathogenesis

Correction to: *Nature* 10.1038/s41586-022-04462-1 Published online 1 February 2022

In the version of this article initially published, there were presentation errors in Extended Data Fig. 7, where the first and third “Delta” images were swapped, while the first Omicron image was an inadvertent duplicate of the original first Delta image. The figure has been replaced in the HTML and PDF versions of the article.

