# Supplementary material for: Attenuated fusogenicity and pathogenicity of SARS-CoV-2 Omicron variant
Source: Nature. 2022 Feb 1;603(7902):700–5. doi: 10.1038/s41586-022-04462-1 (PMC8942852; doi:10.1038/s41586-022-04462-1)
Supplement: Supplementary file 1 — Supplementary Fig. 1 shows the gating strategy for flow cytometry of S-protein-expressing cells and Supplementary Fig. 2 contains the original blots. [file 41586_2022_4462_MOESM1_ESM.pdf]

nature portfolio

https://doi.org/10.1038/s41586-022-04462-1

Supplementary information

Attenuated fusogenicity and pathogenicity of SARS-CoV-2 Omicron variant

In the format provided by the authors and unedited

Supplementary Information

Attenuated fusogenicity and pathogenicity of SARS-CoV-2 Omicron variant

Rigel Suzuki, Daichi Yamasoba, Izumi Kimura, Lei Wang, Mai Kishimoto, Jumpei Ito, Yuhei Morioka, Naganori Nao, Hesham Nasser, Keiya Uriu, Yusuke Kosugi, Masumi Tsuda, Yasuko Orba, Michihito Sasaki, Ryo Shimizu, Ryoko Kawabata, Kumiko Yoshimatsu, Hiroyuki Asakura, Mami Nagashima, Kenji Sadamasu, Kazuhisa Yoshimura, The Genotype to Phenotype Japan (G2P-Japan) Consortium, Hirofumi Sawa, Terumasa Ikeda, Takashi Irie, Keita Matsuno, Shinya Tanaka, Takasuke Fukuhara, Kei Sato

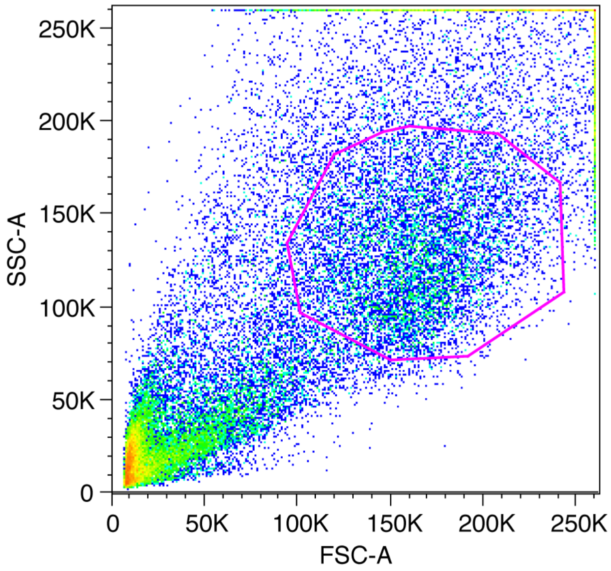

Supplementary Fig. 1. Gating strategy for flow cytometry of S protein-expressing cells.

A representative gating strategy for flow cytometry of S protein-expressing cells (**Fig. 2e, f**) is shown.

Fig. 2h

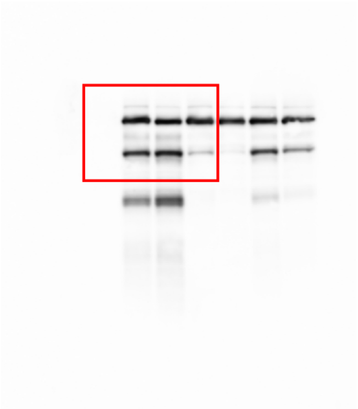

Mouse anti-SARS-CoV-2 S
monoclonal antibody
(clone 1A9, GeneTex,
Cat# GTX632604,
1:10,000)

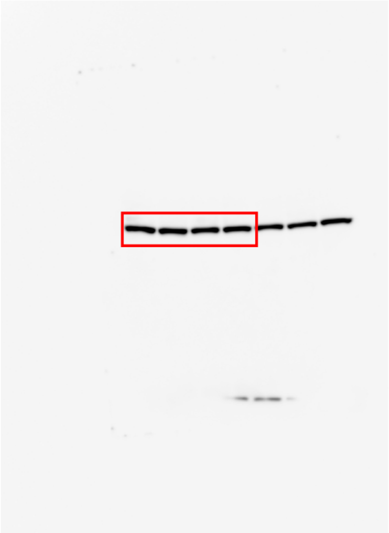

Rabbit anti-beta actin (ACTB)
monoclonal antibody
(clone 13E5, Cell Signalling,
Cat# 4970, 1:5,000)

Fig. 2i

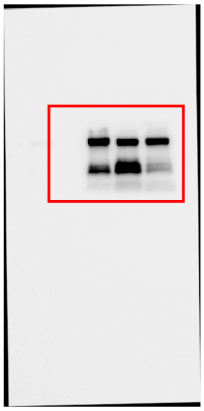

Mouse anti-SARS-CoV-2 S
monoclonal antibody
(clone 1A9, GeneTex,
Cat# GTX632604,
1:10,000)

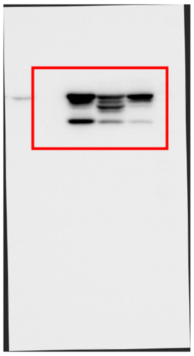

Rabbit anti-SARS-CoV-2 N
monoclonal antibody
(clone HL344, GeneTex,
Cat# GTX635679,
1:5,000)

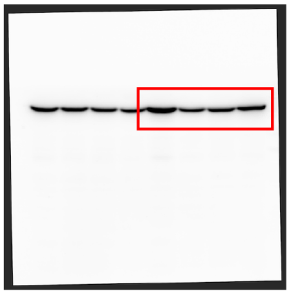

Mouse anti-alpha tubulin (TUBA)
monoclonal antibody
(clone DM1A, Sigma-Aldrich,
Cat# T9026,
1:10,000)

Supplementary Fig. 2. Original (uncrossed) blots.
Uncrossed blots of **Fig. 2h** (top) and **Fig. 2i** (bottom) are shown. Red boxes indicate the cropped area.
