# Supplementary material for: Attenuated fusogenicity and pathogenicity of SARS-CoV-2 Omicron variant
Source: Nature. 2022 Feb 1;603(7902):700–5. doi: 10.1038/s41586-022-04462-1 (PMC8942852; doi:10.1038/s41586-022-04462-1)
Supplement: Supplementary file 2 — Reporting Summary [file 41586_2022_4462_MOESM2_ESM.pdf]

nature portfolio

Corresponding author(s): Matsuno, Tanaka, Fukuhara, Sato

Last updated by author(s): January 8, 2022

Reporting Summary

Nature Portfolio wishes to improve the reproducibility of the work that we publish. This form provides structure for consistency and transparency in reporting. For further information on Nature Portfolio policies, see our Editorial Policies and the Editorial Policy Checklist.

Statistics

For all statistical analyses, confirm that the following items are present in the figure legend, table legend, main text, or Methods section.

n/a	Confirmed	
☐	☒	The exact sample size (*n*) for each experimental group/condition, given as a discrete number and unit of measurement
☐	☒	A statement on whether measurements were taken from distinct samples or whether the same sample was measured repeatedly
☐	☒	The statistical test(s) used AND whether they are one- or two-sided<br>*Only common tests should be described solely by name; describe more complex techniques in the Methods section.*
☒	☐	A description of all covariates tested
☒	☐	A description of any assumptions or corrections, such as tests of normality and adjustment for multiple comparisons
☐	☒	A full description of the statistical parameters including central tendency (e.g. means) or other basic estimates (e.g. regression coefficient) AND variation (e.g. standard deviation) or associated estimates of uncertainty (e.g. confidence intervals)
☐	☒	For null hypothesis testing, the test statistic (e.g. *F*, *t*, *r*) with confidence intervals, effect sizes, degrees of freedom and *P* value noted<br>*Give P values as exact values whenever suitable.*
☐	☒	For Bayesian analysis, information on the choice of priors and Markov chain Monte Carlo settings
☒	☐	For hierarchical and complex designs, identification of the appropriate level for tests and full reporting of outcomes
☒	☐	Estimates of effect sizes (e.g. Cohen's *d*, Pearson's *r*), indicating how they were calculated

Our web collection on statistics for biologists contains articles on many of the points above.

Software and code

Policy information about availability of computer code

Data collection	All-in-One Fluorescence Microscope BZ-X800 (Keyence)<br>QuantStudio 3 Real-Time PCR system (Thermo Fisher Scientific)<br>CFX Connect Real-Time PCR Detection system (Bio-Rad)<br>Eco Real-Time PCR System (Illumina)<br>qTOWER3 G Real-Time System (Analytik Jena)<br>7500 Real-Time PCR System (Thermo Fisher Scientific)<br>Centro XS3 LB960 (Berthhold Technologies)<br>FACS Canto II (BD Biosciences)<br>Amersham Imager 600 (GE Healthcare)<br>FinePointe Station and Review softwares v2.9.2.12849 (STARR)<br>MouseOx PLUS (STARR)<br>Autostainer Link 48 (Dako)<br>MiSeq (Illumina)
Data analysis	CmdStan v2.28.1 (https://mc-stan.org/users/interfaces/cmdstan)<br>cmdstanr v0.4.0 (https://mc-stan.org/cmdstanr/)<br>R v3.6.3 (https://www.r-project.org/)<br>Fiji software v2.2.0 (ImageJ)<br>Sequencher software v5.1 (Gene Codes Corporation)<br>FlowJo software v10.7.1 (BD Biosciences)<br>Image Studio Lite v5.2 (LI-COR Biosciences)<br>NDRscan3.2 software (Hamamatsu Photonics)<br>fastp v0.21.0 [Chen, et al. Bioinformatics 34, i884-i890 (2018). doi:10.1093/bioinformatics/bty560]<br>BWA-MEM v0.7.17 [Li & Durbin. Bioinformatics 26, 589-595 (2010). doi:10.1093/bioinformatics/btp698]

SAMtools v1.9 [Danecek et al. Gigascience 10 (2021). doi:10.1093/gigascience/giab008]
snpEff v5.0e [Cingolani et al. Fly (Austin) 6, 80-92 (2012) doi:10.4161/fly.19695]
Excel software v16.16.8 (Microsoft)
Prism 9 software v9.1.1 (GraphPad Software)
The computational code to estimate the viral transmissibility (Fig. 1) is available in the GitHub repository (https://github.com/TheSatoLab/Estimation_of_transmissibility_of_each_viral_lineage).

For manuscripts utilizing custom algorithms or software that are central to the research but not yet described in published literature, software must be made available to editors and reviewers. We strongly encourage code deposition in a community repository (e.g. GitHub). See the Nature Portfolio guidelines for submitting code & software for further information.

Data

Policy information about availability of data

All manuscripts must include a data availability statement. This statement should provide the following information, where applicable:

- Accession codes, unique identifiers, or web links for publicly available datasets
- A description of any restrictions on data availability
- For clinical datasets or third party data, please ensure that the statement adheres to our policy

The raw data of virus sequences analysed in this study are deposited in Gene Expression Omnibus (accession number: GSE192472). All databases/datasets used in this study are available from GISAID database (https://www.gisaid.org), Genbank database (https://www.ncbi.nlm.nih.gov/genbank/), Our World in Data (https://ourworldindata.org/covid-cases), or outbreak.info (https://outbreak.info). The accession numbers of viral sequences used in this study are listed in Method section.

Field-specific reporting

Please select the one below that is the best fit for your research. If you are not sure, read the appropriate sections before making your selection.

☒ Life sciences ☐ Behavioural & social sciences ☐ Ecological, evolutionary & environmental sciences

For a reference copy of the document with all sections, see nature.com/documents/nr-reporting-summary-flat.pdf

Life sciences study design

All studies must disclose on these points even when the disclosure is negative.

Sample size	The sample sizes (n > 3) for cell culture experiments were chosen for applying statistical tests. The sample sizes (n > 3) for the hamster studies were chosen because they have previously been shown to be sufficient to evaluate a significant difference among groups (Belser et al., Nature, 2013; Zhang et al., Science, 2013; Imai et al., Nature Microbiology, 2020; Saito et al., Nature, 2021).
Data exclusions	In Fig. 3d, the SpO2 data of two infected hamsters at 5 d.p.i. were excluded because these hamsters were not restrained by the sedation.
Replication	In vitro experiments representative of at least 2 experiments with multiple samples per time point. In vivo experiments (hamster) utilized multiple animals per group per time point and were from more than single experiment. In vivo experiments were replicated and performed independently. All attempts at replication were successful.
Randomization	No method of randomization was used to determine how the animals were allocated to the experimental groups and processed in this study, because covariates (sex and age) were identical (male, 4 weeks old). For experiments other than animal studies, randomization is not applicable because homogenous materials (i.e., cell lines) were used. Primary human nasal epithelial cells were used in an experiment, but only one donor/batch was used. Therefore, randomization is not applicable.
Blinding	The number of investigators were limited, and most of experiments were performed in high-containment laboratories. Therefore, blinding was not carried out.

Reporting for specific materials, systems and methods

We require information from authors about some types of materials, experimental systems and methods used in many studies. Here, indicate whether each material, system or method listed is relevant to your study. If you are not sure if a list item applies to your research, read the appropriate section before selecting a response.

Materials & experimental systems

n/a	Involved in the study	
☐	☒	Antibodies
☐	☒	Eukaryotic cell lines
☒	☐	Palaeontology and archaeology
☐	☒	Animals and other organisms
☒	☐	Human research participants
☒	☐	Clinical data
☒	☐	Dual use research of concern

Methods

n/a	Involved in the study	
☒	☐	ChIP-seq
☐	☒	Flow cytometry
☒	☐	MRI-based neuroimaging

Antibodies

Antibodies used

Western blot:
mouse anti-SARS-CoV-2 S monoclonal antibody (clone 1A9, GeneTex, Cat# GTX632604, 1:10,000)
rabbit anti-SARS-CoV-2 N monoclonal antibody (clone HL344, GeneTex, Cat# GTX635679, 1:5,000)
rabbit anti-beta actin (ACTB) monoclonal antibody (clone 13E5, Cell Signalling, Cat# 4970, 1:5,000)
mouse anti-alpha tubulin (TUBA) monoclonal antibody (clone DM1A, Sigma-Aldrich, Cat# T9026, 1:10,000)
HRP-conjugated donkey anti-rabbit IgG polyclonal antibody (Jackson ImmunoResearch, Cat# 711-035-152, 1:10,000)
HRP-conjugated donkey anti-mouse IgG polyclonal antibody (Jackson ImmunoResearch, Cat# 715-035-150, 1:10,000)
For immunofluorescence staining:
rabbit anti-SARS-CoV-2 N polyclonal antibody (GeneTex, Cat# GTX135570, 1:1,000)
Alexa 488-conjugated anti-rabbit IgG antibody (Thermo Fisher Scientific, Cat# A-11008, 1:1,000)
Flow cytometry:
rabbit anti-SARS-CoV-2 S S1/S2 polyclonal antibody (Thermo Fisher Scientific, Cat# PA5-112048, 1:100)
mouse anti-SARS-CoV-2 S monoclonal antibody (clone 1A9, GeneTex, Cat# GTX632604, 1:100)
Normal rabbit IgG (SouthernBiotech, Cat# 0111-01, 1:100)
purified mouse IgG1 isotype control antibody (clone MG1-45, BioLegend, Cat# 401401, 1:100)
APC-conjugated goat anti-mouse or rabbit IgG polyclonal antibody (Jackson ImmunoResearch, Cat# 115-136-146, 1:50 or Cat# 111-136-144, 1:50)
IHC:
mouse anti-SARS-CoV-2 N monoclonal antibody (R&D systems, Clone 1035111, Cat# MAB10474-SP, 1:400)

Validation

Validation of all primary antibodies for the species and application was conducted by manufacturers prior to sale, and validation statements are available on the manufacturers' website.

Eukaryotic cell lines

Policy information about cell lines

Cell line source(s)

HEK293 cells (a human embryonic kidney cell line; ATCC CRL-1573)
HEK293-ACE2/TMPRSS2 cells [HEK293 cells (ATCC CRL-1573) stably expressing human ACE2 and TMPRSS2; Motozono et al., Cell Host & Microbe, 2021)
A549 cells (a human lung epithelial cell line; ATCC CCL-185)
A549-ACE2 cells [A549 cells (ATCC CCL-185) stably expressing human ACE2; Motozono et al., Cell Host & Microbe, 2021]
Vero cells [an African green monkey (Chlorocebus sabaeus) kidney cell line; JCRB0111]
VeroE6/TMPRSS2 cells (JCRB1819)
Calu-3 cells (a human lung epithelial cell line; ATCC HTB-55)
Calu-3/DSP1-7 cells [Calu-3 cells (ATCC HTB-55) stably expressing DSP1-7; Yamamoto et al., Viruses, 2020)
HeLa-ACE2/TMPRSS2 cells [HeLa229 cells (JCRB9086) stably expressing human ACE2 and TMPRSS2; Kawase et al., Journal of Virology, 2012]
Primary human nasal epithelial cells (Cat# EP02, Batch# MP0010, Epithelix)
Vero cells, VeroE6/TMPRSS2 cells, and HeLa-ACE2/TMPRSS2 cells are commercially available at JCRB Cell Bank (https://cellbank.nibiohn.go.jp/english/). Primary human nasal epithelial cells were purchased from Epithelix.

Authentication

None of the cells used were authenticated.

Mycoplasma contamination

All cell lines were regularly tested for mycoplasma contamination by using PCR and were confirmed to be mycoplasma-free.

Commonly misidentified lines
(See ICLAC register)

No commonly misidentified cell lines were used.

Animals and other organisms

Policy information about studies involving animals; ARRIVE guidelines recommended for reporting animal research

Laboratory animals

Syrian hamsters (male, 4 weeks old) were purchased from Japan SLC Inc. (Shizuoka, Japan).

Wild animals

No wild animal was used in this study.

Field-collected samples	No field collected sample was used in the study.
Ethics oversight	All experiments with hamsters were performed in accordance with the Science Council of Japan's Guidelines for Proper Conduct of Animal Experiments. The protocols were approved by the Institutional Animal Care and Use Committee of National University Corporation Hokkaido University (approval numbers 20-0123 and 20-0060).

Note that full information on the approval of the study protocol must also be provided in the manuscript.

Flow Cytometry

Plots

Confirm that:

☒ The axis labels state the marker and fluorochrome used (e.g. CD4-FITC).

☒ The axis scales are clearly visible. Include numbers along axes only for bottom left plot of group (a 'group' is an analysis of identical markers).

☒ All plots are contour plots with outliers or pseudocolor plots.

☒ A numerical value for number of cells or percentage (with statistics) is provided.

Methodology

Sample preparation	HEK293 cells were cotransfected with S expression plasmids (400 ng) and pDSP1-7 (400 ng) using TransIT-LT1 (Takara, Cat# MIR2300).
Instrument	FACS Canto II instrument (BD Biosciences)
Software	FlowJo software v10.7.1 (BD Biosciences)
Cell population abundance	10,000 cells gated in the FSC-A/SSC-A plot (Supplementary Fig. 1) were acquired for each condition.
Gating strategy	Live cell population was gated based on the FSC-A/SSC-A plot. The starting gating strategy is shown in Supplementary Fig. 1. Then, to define the boundary between "positive" and "negative" staining of the surface S protein, isotope control IgG was used instead of primary anti-S antibodies. Grey histograms in Fig. 2e and 2f indicate isotype controls of the assay.

☒ Tick this box to confirm that a figure exemplifying the gating strategy is provided in the Supplementary Information.
