# Supplementary material for: Attenuated fusogenicity and pathogenicity of SARS-CoV-2 Omicron variant
Source: Nature. 2022 Feb 1;603(7902):700–5. doi: 10.1038/s41586-022-04462-1 (PMC8942852; doi:10.1038/s41586-022-04462-1)
Supplement: Supplementary file 3 — Primers used for the construction of Omicron S expression plasmid. [file 41586_2022_4462_MOESM3_ESM.pdf]

**Supplementary Table 1. Mutations in Delta and Omicron isolates.**

| Mutations detected in a Delta isolate (B.1.617.2 lineage, strain TKYTK1734, GISAID ID: EPI_ISL_2378732) compared to an original SARS-CoV-2 (B lineage, strain Wuhan-Hu-1, GenBank accession no. NC_045512.2) | | | | | |
|---|---|---|---|---|---|
| Nucleotide position | Reference | Mutation | Mutation type | ORF | Amino acid substitution |
| 210 | G | T | upstream_gene_variant | NSP1 | |
| 241 | C | T | upstream_gene_variant | NSP1 | |
| 1191 | C | T | missense_variant | NSP2 | P129L |
| 1267 | C | T | synonymous_variant | NSP2 | G154G |
| 3037 | C | T | synonymous_variant | NSP3 | F106F |
| 5184 | C | T | missense_variant | NSP3 | P822L |
| 9891 | C | T | missense_variant | NSP4 | A446V |
| 11418 | T | C | missense_variant | NSP6 | V149A |
| 12102 | C | T | missense_variant | NSP8 | S4L |
| 12946 | T | C | synonymous_variant | NSP9 | Y87Y |
| 14408 | C | T | missense_variant | NSP12 | P303L |
| 15451 | G | A | missense_variant | NSP12 | G651S |
| 16466 | C | T | missense_variant | NSP13 | P77L |
| 18176 | C | T | missense_variant | NSP14 | P46L |
| 20148 | C | T | synonymous_variant | NSP15 | F176F |
| 20262 | A | G | synonymous_variant | NSP15 | L214L |
| 21618 | C | G | missense_variant | S | T19R |
| 21987 | G | A | missense_variant | S | G142D |
| 22028 | GAGTTCA | G | disruptive_inframe_deletion | S | EFR156-158G |
| 22917 | T | G | missense_variant | S | L452R |
| 22995 | C | A | missense_variant | S | T478K |
| 23403 | A | G | missense_variant | S | D614G |
| 23604 | C | G | missense_variant | S | P681R |
| 24410 | G | A | missense_variant | S | D950N |
| 25469 | C | T | missense_variant | ORF3a | S26L |
| 26767 | T | C | missense_variant | M | I82T |
| 27638 | T | C | missense_variant | ORF7a | V82A |
| 27739 | C | T | missense_variant | ORF7a | L116F |
| 27752 | C | T | missense_variant | ORF7a | T120I |
| 28247 | AGATTTC | A | conservative_inframe_deletion | ORF8 | DF119-120del |
| 28270 | TA | T | upstream_gene_variant | N | |
| 28461 | A | G | missense_variant | N | D63G |
| 28881 | G | T | missense_variant | N | R203M |
| 29402 | G | T | missense_variant | N | D377Y |
| 29742 | G | T | downstream_gene_variant | | |

| Mutations detected in an Omicron isolate (BA.1 lineage, strain TY38-873, GISAID ID: EPI_ISL_7418017) compared to an original SARS-CoV-2 (B lineage, strain Wuhan-Hu-1, GenBank accession no. NC_045512.2) | | | | | |
|---|---|---|---|---|---|
| Nucleotide position | Reference | Mutation | Mutation type | ORF | Amino acid substitution |
| 241 | C | T | upstream_gene_variant | NSP1 | |
| 2832 | A | G | missense_variant | NSP3 | K38R |
| 3037 | C | T | synonymous_variant | NSP3 | F106F |
| 5386 | T | G | synonymous_variant | NSP3 | A889A |
| 5673 | C | T | missense_variant | NSP3 | P985L |
| 5730 | C | T | missense_variant | NSP3 | T1004I |
| 6512 | AGTT | A | disruptive_inframe_deletion | NSP3 | SL1265-1266I |
| 8393 | G | A | missense_variant | NSP3 | A1892T |
| 10029 | C | T | missense_variant | NSP4 | T492I |
| 10447 | GCC | GTA | missense_variant | NSP5 | P132Y |
| 11282 | AGTTTGTCTG | A | disruptive_inframe_deletion | NSP6 | LSG105-107del |
| 11537 | A | G | missense_variant | NSP6 | I189V |
| 13195 | T | C | synonymous_variant | NSP10 | V57V |
| 14408 | C | T | missense_variant | NSP12 | P303L |
| 15240 | C | T | synonymous_variant | NSP12 | N580N |
| 18163 | A | G | missense_variant | NSP14 | I42V |
| 21762 | C | T | missense_variant | S | A67V |
| 21764 | ATACATG | A | disruptive_inframe_deletion | S | HV69-70del |
| 21846 | C | T | missense_variant | S | T95I |
| 21986 | GGTGTTTATT | G | disruptive_inframe_deletion | S | GVYY142-145D |
| 22193 | AATT | A | disruptive_inframe_deletion | S | NL211-212I |
| 22204 | T | TGAGCCAGAA | disruptive_inframe_insertion | S | RD214-215EPE |
| 22578 | G | A | missense_variant | S | G339D |
| 22672 | TTC | TCT | missense_variant | S | S371L |
| 22679 | T | C | missense_variant | S | S373P |
| 22686 | C | T | missense_variant | S | S375F |
| 22813 | G | T | missense_variant | S | K417N |
| 22882 | T | G | missense_variant | S | N440K |
| 22898 | G | A | missense_variant | S | G446S |
| 22992 | G | A | missense_variant | S | S477N |
| 22995 | C | A | missense_variant | S | T478K |
| 23013 | A | C | missense_variant | S | E484A |
| 23040 | A | G | missense_variant | S | Q493R |
| 23048 | G | A | missense_variant | S | G496S |
| 23055 | A | G | missense_variant | S | Q498R |
| 23063 | A | T | missense_variant | S | N501Y |
| 23075 | T | C | missense_variant | S | Y505H |
| 23202 | C | A | missense_variant | S | T547K |
| 23403 | A | G | missense_variant | S | D614G |
| 23525 | C | T | missense_variant | S | H655Y |
| 23599 | T | G | missense_variant | S | N679K |
| 23604 | C | A | missense_variant | S | P681H |
| 23854 | C | A | missense_variant | S | N764K |
| 23948 | G | T | missense_variant | S | D796Y |
| 24130 | C | A | missense_variant | S | N856K |
| 24424 | A | T | missense_variant | S | Q954H |
| 24469 | T | A | missense_variant | S | N969K |
| 24503 | C | T | missense_variant | S | L981F |
| 25000 | C | T | synonymous_variant | S | D1146D |
| 25584 | C | T | synonymous_variant | ORF3a | T64T |
| 26270 | C | T | missense_variant | E | T9I |
| 26530 | A | G | missense_variant | M | D3G |
| 26577 | C | G | missense_variant | M | Q19E |
| 26709 | G | A | missense_variant | M | A63T |
| 27259 | A | C | synonymous_variant | ORF6 | R20R |
| 27807 | C | T | synonymous_variant | ORF7b | L18L |
| 28271 | A | T | upstream_gene_variant | N | |
| 28311 | C | T | missense_variant | N | P13L |
| 28361 | GGAGAACGCA | G | disruptive_inframe_deletion | N | ERS31-33del |
| 28880 | AGGG | AAAC | missense_variant | N | RG203-204KR |
| 29645 | G | T | missense_variant | ORF10 | V30L |
